# Health characteristics and factors associated with transition shock in newly graduated registered nurses: a latent class analysis

**DOI:** 10.1186/s12912-024-01862-8

**Published:** 2024-03-27

**Authors:** Pan Wang, Juan Zhou, Xin Shen, Yaping Ge, Yanran LI, Hui Ge, Shuwen LI

**Affiliations:** 1 Department of cardiac surgery, Zhong Da Hospital, Southeast University, Dingjiaqiao NO.87, Nanjing, 210009 China; 2 School of nursing, Anhui Medical University, Hefei, Feicui Road NO.15, Hefei, 230032 China

**Keywords:** Transition shock, Newly graduated registered nurses, Latent class analysis

## Abstract

**Background:**

Transition shock occurs at a vulnerable time in newly graduated registered nurses’ careers and has a clear impact on both newly graduated registered nurses’ productivity and patient recovery outcomes. Identifying classification features of transition shock and targeting interventions to support newly graduated registered nurses is imperative. The study aimed to explore potential transition shock subgroups of newly graduated registered nurses and further explore the impact of population characteristics and two indices of health on transition shock.

**Methods:**

A descriptive, cross-sectional design was conducted. An online questionnaire was sent via WeChat to newly graduated registered nurses who started work in 2021 at seven hospitals between August and November 2021, and 331 nurses filled out the questionnaire. Latent class analysis was used to identify the potential class of the transition shock of newly graduated registered nurses, and multinomial logistic regression analyses were used to determine the factors of potential classification.

**Results:**

The study identified four classes of transition shock in newly graduated registered nurses, namely, “high transition shock”, “physical fatigue-lack of knowledge”, “development adaptation” and “low transition shock-worry” groups. Newly graduated registered nurses who urinated less than 4 times per day (OR = 0.051, 95% CI = 0.005–0.502) were likely to be in the “high transition shock” group. Newly graduated registered nurses who did not delay urination (OR = 4.267, 95% CI = 1.162–11.236) were more likely to belong to the “low transition shock-worry” group. Newly graduated registered nurses without sleep disturbance were more likely to be in the “physical fatigue - lack of knowledge” (OR = 3.109, 95% CI = 1.283–7.532), “development adaptation” (OR = 8.183, 95% CI = 2.447–27.066), and “low transition shock-worry” (OR = 8.749, 95% CI = 1.619–47.288) groups than in the ‘high transition shock’ group.

**Conclusions:**

This study highlights potential patterns of transition shock among newly graduated registered nurses. Two indices of health, namely, delayed urination and sleep disturbance, can predict the subgroups of newly graduated registered nurses with transition shock.

## Introduction

Globally, the demand for nurses has risen rapidly due to innovative developments in medical technology and increased demand for services related to an aging population and chronic and noncommunicable diseases. Nursing education in China has developed rapidly over the past decade, and a large number of nurses have been educated, which can theoretically meet the growing demand for healthcare. However, You [1] reported that the number of nursing graduates has grown rapidly, but the number of registered nurses has not increased by an equal amount in the same year, which was closely related to the high turnover rate of nurses. With the increase in multilevel and diversified health service demand, more attention should be placed on attracting and retaining nurses to alleviate the ongoing crisis of nurse shortages in the workplace.

Notably, newly graduated registered nurses within a year of graduation reported a higher turnover rate than experienced nurses, and the turnover rate of new nurses (29.0%) was 2.1 times higher than that of experienced nurses (13.9%) [2]. A survey on the actual turnover rate in China showed that the turnover rate of nurses who have worked for less than 1 year (4.87%) is 6.6 times higher than that of nurses who have worked for more than 10 years (0.74%). Harrowing, a longitudinal study in China, showed that 71.8% of newly graduated registered nurses intended to leave in the first year of their job [3].

## Background

Transition shock is defined as a challenging and intimidating process with a broad scope of physical, emotional, developmental, and intellectual changes when newly graduated registered nurses engage in a professional practice position for the first time [4, 5]. Previous studies have indicated that a high level of transition shock is the main reason why newly graduated registered nurses consider leaving in the first year [3, 6]. Transition shock affects newly graduated registered nurses’ physical and psychological health, resulting in low work satisfaction [2], work adjustment disorder [7], and work-life imbalance [8] and ultimately leading them to leave the nursing profession. Furthermore, high levels of transition shock affect nurse productivity, as well as the quality of care provided to patients, leading to worse recovery outcomes [9].

Previous studies used quantitative research to investigate the current state of transition shock or qualitative research to explore the experience of transition for newly graduated registered nurses. A study conducted by Kim [10] found that newly graduated registered nurses reported high levels of transition shock, similar to another Chinese study [11]. Qualitative studies have shown that the transition from student to nurse roles can be a stressful and challenging process [8, 12]. However, no studies indicated the cutoff scores that distinguish between high, medium, and low levels of transition shocks, and no relevant references are provided. The inability to effectively distinguish between types of transition shocks in newly graduated registered nurses makes precision intervention difficult. In addition, the variable-centered approach simply regarded transition shocks as a whole and used the total score to judge the level of transition shock. Even if two newly graduated registered nurses had the same score on the scale, they may respond differently on different items, so this approach ignores individual differences within the group [13].

For nursing managers, the prerequisite for achieving precise interventions is the accurate delineation of the level of transition shock. With the rapid development of quantitative research methods in the social sciences, the person-centered approach has gradually attracted the attention of researchers. Compared with the traditional variable-centered approach, Latent Class Analysis (LCA) can judge the latent classification of individuals based on their response patterns to manifest variables and divide the proportion of different classes in the overall population to further capture the characteristics and inequality of different types of populations [14]. Thus, the objective of this study was to use LCA to describe potential patterns of transition shock among newly graduated registered nurses, which could provide useful insights for nursing managers when developing tailored interventions to support the transition from student to nurse.

Health characteristics are important determinants of work adjustment and recovery outcomes and are likely to brunt on nurse retention [15]. A prospective longitudinal study [16] found that an unhealthy lifestyle, such as poor sleep quality of newly graduated registered nurses, was a risk factor for turnover. Another study found that newly graduated registered nurses with sleep disturbances may be more susceptible to stressful stimuli and lack work resilience [17]. In Xu’s study [18], nurses often engage in unhealthy toileting behaviors such as delayed urination to adapt to occupational stress. Increased work burden was in turn found to be associated with the number of breaks and limitations to restroom use, which led to increased delayed urination behavior [19]. Strong evidence for the relationship between sleep, delayed urination, and transition shock is still lacking. Thus, the second objective of the current study was to explore the impact of health characteristics on transition shock, specifically sleep and delayed urination.

## Methods

### Aim

This study aimed to explore potential subgroups of newly graduated registered nurses’ transition shock and further explore the population characteristics and two indices of health, namely, urination and sleep disturbance, of different subgroups. This study provides a reference for the formulation of targeted intervention strategies to reduce the transition shock of newly graduated registered nurses and ultimately improve the quality of nursing.

### Design

A descriptive, cross-sectional design was conducted.

### Participants and settings

Newly graduated registered nurses from seven hospitals were recruited in H City, China. In the Chinese medical system, hospitals are divided into three levels according to their functions and tasks. A primary hospital is a grassroots hospital that directly provides preventive, medical, health care, and rehabilitation services to communities of a certain population. The secondary hospital provides comprehensive medical and health services to multiple communities and undertakes certain teaching and scientific research tasks. The tertiary hospital provides medical and health services to the whole country and is a medical prevention technology center with comprehensive medical, teaching, and scientific research capabilities. For newly graduated registered nurses, secondary hospitals and tertiary hospitals have better salaries and development prospects, and they are more likely to choose to work in secondary and tertiary hospitals [20]. Therefore, the hospitals selected in this study were all secondary or tertiary. Newly graduated registered nurses meeting the following criteria were included: (1) registered nurses; (2) worked directly in the hospital after graduation; and (3) had less than one year of work experience.

### Sample size

The formula for the sample size calculation used in this study was N = (U_a/2_*S/δ)^2^, assuming that α = 0.05 and U_a/2_ = 1.96. According to Cao et al.’s [21] research,‾x = 2.83, S = 0.84, and a total of 225 newly graduated registered nurses were needed. A total of 321 newly graduated registered nurses were deemed appropriate to participate in this study, taking into account the 30% nonresponse or missing data. Eventually, 355 newly graduated registered nurses were asked to take part in the study using convenience sampling, and 331 of them responded (93.24% response rate).

### Data collection

The data were collected from August to November 2021 through online questionnaires distributed by WENJUANXING (www.wjx.cn), a professional online questionnaire survey platform. A link or QR code to the questionnaire was sent to the trained administrators of each hospital, who forwarded it to the online management WeChat groups. The objectives, procedures, and anonymity of this study were explained to all participants before the investigation, and informed consent was obtained from the participants. Newly graduated registered nurses were required to complete the questionnaire individually within a week.

### Measures

#### Demographic characteristics

The demographic data collected in this study included age, sex, education level, family situation, shift work, body mass index (BMI), urination behavior, and sleep.

#### Transition shock

Transition shock was evaluated by the Chinese version of the Transition Shock of Newly Graduated Nurses Scale [22], a self-reported tool that comprises 27 items divided into 4 dimensions: physical, psychological, knowledge and skills, and social culture and development. A 5-point Likert scale was used to assess each item ranging from 1 = “totally disagree” to 5 = “totally agree”, with higher scores indicating more serious transition shock. The Cronbach’s α was 0.918, and in this study, it was 0.952.

The Chinese versions of the scales selected in this study are widely used by nurses and have achieved good reliability and validity (see measure described above). Good reliability and validity were also reported in this study. To reduce potential bias, investigators who were responsible for data analysis did not participate in the distribution and collection of the questionnaires. The administrators did not know the newly graduated registered nurses’ participation or their ratings of the questionnaires. Several quality control methods were employed to reduce bias during the study design, data collection, and data analysis and to control for confounding bias by considering covariates wherever possible.

### Ethical considerations

The study was conducted following the Helsinki Declaration and was approved by the ethics committee of Anhui medical university and its affiliated hospitals (YX2021-090). In the beginning of the study, an email with detailed information about the study objectives, procedures, and informed consent form was sent to all participants, and they were requested to return the signed informed consent form. Data were encrypted and stored in a safe place and were only used for this research.

### Data analysis

Mplus 8.3 software and SPSS 23.0 statistics program were used for data analysis. Statistical significance was set at a two-tailed 5% level. An original score of more than or equal to 3 is a high response probability, which is recorded as 1 point; an original score of less than 3 is a low response probability, which is recorded as 0 points, and then the transformed data are subjected to LCA. LCA was conducted to identify the latent classes of newly graduated registered nurses in transition shocks. Starting from the initial model, 1 to 5 latent class models were established gradually, and the best model was selected when the fitness indices reached the optimal level. Classification accuracy was assessed by fitness indices including Akaike Information Criterion (AIC), Bayesian Information Criterion (BIC), and the sample size adjusted Bayesian information criterion (aBIC), Entropy Index, and Likelihood Ratio (LMR), Bootstrap Likelihood Ratio TEST (BLRT), etc. Among them, the smaller the AIC and BIC and aBIC, the better model fit. The entropy value is between 0 and 1; the closer to 1, the higher the classification accuracy. The LMR and BLRT tests have a statistically significant difference (*p* <.05), indicating that the K model is better than K-1. The statistical descriptions used mean ± standard deviation, or frequency and composition ratio. Subgroup demographic characteristics were explored using chi-square tests, Fisher’s exact test, and analysis of variance. In the analysis of the factors influencing the transition shock, the level of the transition shock is used as the dependent variable (control = “high transition shock”), and the number of levels is greater than 2, and *p* < 0. 05 in the test of parallel lines, so multinomial logistic regression analysis was used.

## Results

A total of 355 questionnaires were distributed, 331 electronic questionnaires were recovered, and 24 did not respond, with an effective recovery rate of 93.2%. Among the 331 participants, there were 40 males (12.1%) and 291 females (87.9%). The ages ranged from 18 to 33, with an average age of 22.41 ± 1.47 years, and 239 (72.7%) of them had a bachelor’s degree or above. The total scores for transition shock and its 4 dimensions: physical, psychological, knowledge and skills, and social culture and development were 74.92(19.27), 18.97(5.16), 21.98(6.54), 14.91(4.20), and 19.07(6.18), respectively. (Table 1)

**Table 1:** Comparison of 4 classes of transition shock scores in each dimension (*n* = 331)(M ± SD)

Variable	Overall (*n* = 331)	C1 (*n* = 78)	C2 (*n* = 122)	C3 (*n* = 88)	C4 (*n* = 43)	F	p
physical	18.97 ± 5.16	23.14 ± 3.49	20.62 ± 3.59	16.27 ± 3.87	12.21 ± 4.03	104.553	< 0.001
psychological	21.98 ± 6.54	29.15 ± 4.42	23.46 ± 3.76	17.75 ± 3.49	13.40 ± 3.34	194.049	< 0.001
knowledge and skills	14.91 ± 4.20	18.44 ± 3.27	16.42 ± 2.59	12.76 ± 2.55	8.60 ± 2.50	148.820	< 0.001
social culture and development	19.07 ± 6.17	26.19 ± 4.64	19.66 ± 3.97	15.36 ± 3.59	12.02 ± 3.58	153.153	< 0.001
Transition shock (total)	74.92 ± 19.27	96.92 ± 12.38	80.16 ± 8.57	62.15 ± 7.31	46.23 ± 12.29	312.709	< 0.001

Abbreviations: M, mean; SD, standard deviation; C1: high transition shock group; C2: physical fatigue - lack of knowledge group; C3: development adaptation group; C4: low transition shock-worry group

### Latent classes of transition shock

Five latent class models were estimated, and the results were shown in Table 2. From model 1 to model 5, the entropy values were all > 0.8, and the AIC, BIC, and aBIC continued to decrease. A statistically nonsignificant LMR indicated that the 5-class model was not better than the 4-class model. Consequently, the 4-class model was finally determined to be the optimal model.

**Table 2:** Fit indices of each model

Model	AIC	BIC	aBIC	Entropy	LMR	BLRT	Category probability(%)
1	11055.510	11158.168	11072.523				
2	9200.613	9409.730	9235.268	0.935	< 0.001	< 0.001	0.574/0.426
3	8856.884	9172.460	8909.182	0.906	0.002	< 0.001	0.248/0.408/0.344
**4**	**8750.365**	**9172.400**	**8820.305**	**0.892**	**0.023**	**< 0.001**	**0.235/0.266/0.369/0.130**
5	8657.865	9186.360	8745.447	0.894	0.214	< 0.001	0.260/0.130/0.130/0.247/0.233

Abbreviations: AIC, Akaike’s information criterion; BIC, Bayesian information criterion; aBIC, sample-size adjusted BIC; LMR, Lo-Mendell-Rubin; BLRT, bootstrapped parametric likelihood ratio test

Table 3 displays the probabilities of belonging to the four prospective categories. The probabilities of nurses’ transition shock (rows) belonging to each potential category.

(columns), the first row of results shows that 94.1% of individuals were correctly assigned to C1 and only 5.9% of individuals were incorrectly assigned to C3. The probability of category belonging to the four categories range from 93.1 to 95.5% on average, indicating that the results of classifying into four potential categories are reliable.

**Table 3:** Average attribution probability of participants (Rows) in each potential profile (Column)

	C1	C2	C3	C4
C1	0.941	0.000	0.059	0.000
C2	0.000	0.955	0.037	0.009
C3	0.029	0.040	0.931	0.000
C4	0.000	0.065	0.000	0.935

**Fig. 1:**
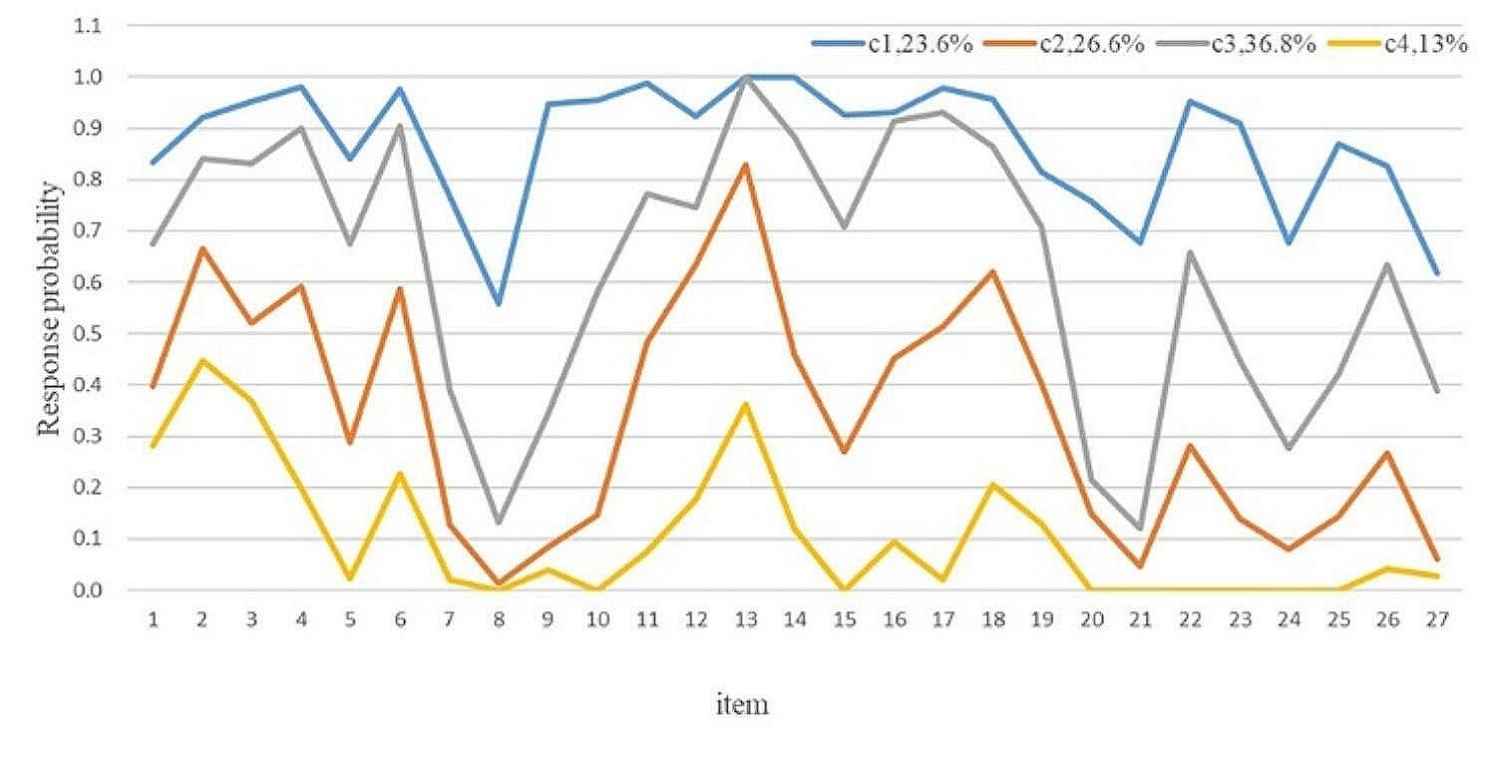
Four subtypes of transition shock based on the LCA results

The scores for the four classes of transition shocks are shown in Fig. 1. C1 had the highest responses on all items, so it was named the “high transition shock” group (23.6%). C2 had a high response in the physical dimension and the knowledge skills dimension of the scale, so it was named the “physical fatigue - lack of knowledge” group (36.8%). C3 has a medium response on all items, and the response probability of the psychological, social culture, and personal development dimensions is relatively low, so it was named the “development adaptation” group (26.6%). C4 the lowest responses on all items, so it was named the “low transition shock-worry” group (13%).

### Differences in the latent classes by characteristics

The distribution characteristics of newly graduated registered nurses in different classes on demographic variables are shown in Table 4. Newly graduated registered nurses in the ‘high transition shock’ group earned the highest scores for transition shock. Newly graduated registered nurses in the ‘physical fatigue - lack of knowledge’ and ‘development adaptation’ groups presented parallel trajectories of transition shock, while newly graduated registered nurses in the ‘low transition shock-worry’ group had the lowest levels of transition shock. Newly graduated registered nurses in the “low transition shock-worry” group had the largest proportion of associate degrees [53.5% vs. 34.6%, 19.7%, 27.3%] and no sleep disturbance [83.7% vs 30.8%, 56.6%, 76.1%]. The “high transition shock” group had the highest rate of holding back urine [84.6% vs. 82%, 69.3%, 51.2%].

**Table 4:** Demographic and health-related characteristics by latent class

	high transition shock (*n* = 78) n(%)	physical fatigue - lack of knowledge (*n* = 122) n(%)	development adaptation (*n* = 88) n(%)	low transition shock-worry (*n* = 43) n(%)	χ^2^/F	p
Gender					2.255	0.521
Female	71 (91)	109 (89.3)	75 (85.2)	36 (83.7)		
Male	7 (9)	13 (10.7)	13 (14.8)	7 (16.3)		
Age(‾x ± s)	22.95 ± 1.77	22.83 ± 1.5	22.86 ± 1.67	22.49 ± 1.88	0.721	0.540
Education level					18.571 ^a^	0.004
Associate degree	21 (34.6)	24 (19.7)	24 (27.3)	23 (53.5)		
Bachelor’s degree	48 (61.5)	88 (72.1)	59 (67)	18 (41.9)		
Master’s degree	9 (11.4)	10 (8.2)	5 (5.7)	2 (4.6)		
Family situation					1.483	0.686
Only child	10 (12.8)	23 (18.9)	15 (17)	6 (14)		
Nononly child	68 (87.2)	99 (81.1)	73 (83)	37 (86)		
Night shift frequency					2.147 ^a^	0.915
0–4 times/month	59 (75.6)	89 (73.0)	59 (67.0)	31 (72.1)		
5–9 times/month	13 (16.7)	25 (20.5)	22 (25.0)	9 (20.9)		
More than 10 times/month	5 (6.4)	8 (6.5)	7 (8.0)	3 (7.0)		
BMI(‾x ± s)	20.33 ± 2.56	20.11 ± 2.09	20.47 ± 2.70	20.61 ± 2.97	0.599 ^b^	0.540
Frequency of urination					19.499	0.002
Less than 4 times	25 (32)	36 (29.5)	9 (10.2)	13 (30.2)		
4 to 8 times	52 (66.7)	81 (66.4)	71 (80.7)	29 (67.5)		
More than 8 times	1 (1.2)	5 (4.1)	8 (9.1)	1 (2.3)		
Hold off pissy					21.673	< 0.001
Yes	66 (84.6)	100 (82)	61 (69.3)	22 (51.2)		
No	12 (15.4)	22 (18)	27 (30.7)	21 (48.8)		
Duration of sleep					18.186 ^a^	0.002
Less than 6 h	41 (52.7)	44 (30.1)	22 (25)	17 (39.5)		
7 to 8 h	36 (46.1)	76 (62.3)	65 (73.9)	23 (53.5)		
More than 8 h	1 (1.2)	2 (1.6)	1 (1.1)	3 (7)		
Disturbance of sleep					49.977 ^a^	< 0.001
Never	24 (30.8)	69 (56.6)	67 (76.1)	36 (83.7)		
Once or twice a week	31 (39.7)	34 (27.9)	16 (18.2)	5 (11.6)		
More than 2 times per week	23 (29.5)	19 (15.5)	5 (5.7)	2 (4.7)		
Quality of sleep					24.593	< 0.001
Good	8 (10.3)	13 (10.7)	15 (17)	9 (20.9)		
Average	42 (53.8)	84 (68.9)	64 (72.7)	31 (72.1)		
Poor	28 (35.9)	25 (20.4)	9 (10.2)	3 (7)		

Abbreviations: a, Fisher’s exact test; b, Analysis of variance

### Predictor of latent class membership

In the parallel test, the *p*-value < 0.05, so Multinomial logistic regression analyses were performed to determine the factors of class membership using the ‘high transition shock’ group as the reference group (Table 5). Newly graduated registered nurses who urinated less than 4 times per day (OR = 0.051, *p* = .011) tended to be in the “high transition shock” group. Newly graduated registered nurses who did not delay urination (OR = 4.267, *p* = .003) were more likely to belong to the “low transition shock-worry” group. Newly graduated registered nurses without sleep disturbance were more likely to be in the “physical fatigue - lack of knowledge” (OR = 3.109, *p* = .012), “development adaptation” (OR = 8.183, *p* = .001), and “low transition shock-worry” (OR = 8.749, *p* = .012) groups than in the ‘high transition shock’ group.

**Table 5:** Multinomial logistic regression results for latent profiles of NGRNs’ transition shock (*N* = 331)

	physical fatigue - lack of knowledge			development adaptation			low transition shock-worry		
	**B**	**OR (95%CI)**	***p***	**B**	**OR (95%CI)**	***p***	**B**	**OR (95%CI)**	***p***
Frequency of urination Less than 4 (cg = more than 8)	-1.054	0.349 (0.037 ~ 3.255)	0.306	-2.979	0.051 (0.005 ~ 0.502)	0.011	-0.491	0.612 (0.030 ~ 12.345)	0.749
Never hold off pissy (cg = hold off pissy)	-0.096	0.908 (0.398 ~ 2.070)	0.722	0.496	1.642 (0.696 ~ 3.871)	0.551	1.451	4.267 (1.621 ~ 11.236)	0.003
No sleep disturbance (cg = more than 2 times per week)	1.134	3.109 (1.283 ~ 7.532)	0.012	2.097	8.138 (2.447 ~ 27.066)	0.001	2.169	8.749 (1.619 ~ 47.288)	0.012

Note: Class “high transition shock” was selected as the reference

Abbreviations: CI, confidence interval; OR, odds ratio; cg, control group

## Discussion

The study identified four classes of transition shock in newly graduated registered nurses, namely, “high transition shock”, “physical fatigue - lack of knowledge”, “development adaptation” and “low transition shock-worry” groups. Newly graduated registered nurses in the high transition shock group scored highest on all items, indicating that they were affected by the transition from all aspects. This was consistent with Duchscher and Graf’s [4, 23] research, which indicated that newly graduated registered nurses experienced drastic changes in responsibility, knowledge, roles, and relationships at the beginning of their careers, resulting in multilevel shocks. In this group, newly graduated registered nurses’ energy was overspent as a direct consequence of excitement and overstimulation, and with negative emotions such as being overwhelmed, scared, self-doubt, and fearful, they were unable to make advanced clinical decisions. Meanwhile, it was also arduous for them to integrate themselves into an unfamiliar but team-oriented environment in a short time [7]. For newly graduated registered nurses in the “high transition shock” group, nursing managers or policymakers should focus on providing transition programs to facilitate the transition of students to clinical nurses [5]. In addition, sufficient supervisor support, appropriate workload delegation, and effective communication correlate with the stage of development of the transition to meet the fluctuating demands of new nurses to reduce transition shock [24].

Newly graduated registered nurses in the “physical fatigue - lack of knowledge” group endorsed a high level of physical and knowledge and skills shock. A possible reason is that there is a certain gap between theoretical knowledge and clinical practice, which makes their reserve of professional knowledge and skills inadequate to meet the real work demands. However, managers expected them to handle the workload as experienced practitioners in a few weeks [25]. To be approved by superiors and colleagues, newly graduated registered nurses tended to choose to focus on completing daily nursing work at the expense of their own rest time. This kept their bodies in a state of ‘perpetual work’, resulting in a series of physical shocks, such as somatic exhaustion [23] and sleep disturbance [17], during the transition period. For newly graduated registered nurses in the “physical fatigue-lack of knowledge” group, it is necessary to change their insufficient knowledge reserve. Similar to the nurse residency program (NRP), a unified training mode for clinical practice ability should be established to improve newly graduated registered nurses’ clinical practice skills and facilitate the transition through a model that combines clinical knowledge instruction and practical training [26]. The researchers recommended the use of diverse training models to help newly graduated registered nurses adapt to clinical work, such as scenario-based simulation. It enables newly graduated registered nurses to engage in experiential learning, which is particularly applicable to the simulation of clinical emergencies, bringing into play the subjective initiative of newly graduated registered nurses to develop their critical thinking skills and clinical practice skills [27].

Newly graduated registered nurses in the “low transition shock-worry” group earned the lowest scores for each item, especially in the sociocultural and personal development dimensions. This result supported the notion that some newly graduated registered nurses show better job adaptation and lower transition shocks than others. A study on the organizational and personal factors of newly graduated registered nurses revealed that they felt a sense of belonging and engagement when their values were aligned with those of the workplace. This was also the reason why they remained within the nursing profession [8]. Another study indicated that optimistic and extroverted nurses were more likely to actively seek solutions to problems and to make adaptive changes to the external environment; however, pessimistic and introverted nurses did the opposite [28]. Despite these studies exploring some features in newly graduated registered nurses with low levels of transition shock, further in-depth studies are required to expound why some newly graduated registered nurses suffer from lower transition shock during the transition period.

Newly graduated registered nurses who urinated less than 4 times a day were more likely to be in the “high transition shock” group than in the “development adaptation” group, and those who did not hold back urine were seemingly in the “low transition shock-worry” group. Delaying voiding is a common practice among nursing staff and may impair productivity, increase working pressure, compromise clinical care, facilitate the development of serious health conditions, and contribute to transition shock [29]. Some measures could be taken to maintain the healthy toilet behavior of newly graduated registered nurses, such as implementing health behavior education and removing workplace barriers to healthy practices (e.g., heavy workload, irregular breaks, inadequate amenities) [30]. Furthermore, they should provide a supportive work environment, maintain regular communication and psychological support, and address specific difficulties and problems at work in a timely manner.

Newly graduated registered nurses without sleep disturbance were less likely to be in the ‘high transition shock’ group. Sleep disturbance has been reported as a prevalent health problem among nurses. A prospective longitudinal study of the association between trajectories of sleep disturbance and turnover rates in the first two years of work for novice nurses indicated that nurses with severely disturbed sleep tend to leave their first organization [31]. This experience contributes to higher levels of transition shock and leads to other problems that influence voluntary job termination. Nursing managers should assess and manage newly graduated registered nurses’ sleep disturbances to ensure dynamism at work, including implementing healthy working schedules, teaching self-care management strategies for sleep difficulties, and advancing health promotion programs [17, 31].

### Implications for research and practice

The current study provides evidential support for the perspective that there are individual differences in the transition shock of newly graduated registered nurses. Nursing managers should implement targeted interventions for different types of transition shocks and should pay particular attention to those who are likely to be in “high transition shock” and “physical fatigue - lack of knowledge” groups. In this study, we identified two indices of health, namely, delayed urination and sleep disturbance, that can predict the subgroup of newly graduated registered nurses’ transition shock. However, without theoretical and empirical evidence, it is unclear whether different levels of transition shock affect the health characteristics of newly graduated registered nurses. Further research is required using a longitudinal design and a larger sample size to assess health characteristics and transition shock for newly graduated registered nurses in the nursing workforce.

### Limitations

This study has several limitations that should be noted. First, the cross-sectional study designs cannot determine causal relationships between variables, and future longitudinal studies should identify the relationship between health characteristics and transition shock. Second, the findings were obtained primarily in a female sample (87.9% of the participants were female), so the conclusions need to be generalized with caution. Last, this is self-reported data and should be identified as a limitation.

## Conclusions

Transition shock occurs at a vulnerable time in newly graduated registered nurses’ careers and has a clear impact on both newly graduated registered nurses’ productivity and patient recovery outcomes. This study identified four potential classes of transition shock: “high transition shock”, “physical fatigue - lack of knowledge”, “development adaptation”, and “low transition shock-worry”. Approximately 60.4% of newly graduated registered nurses were in the first two classes. We identified two indices of health, namely, delayed urination and sleep disturbance, that can predict the subgroup of newly graduated registered nurses’ transition shock. The current data can help nursing managers provide precise interventions to help newly graduated registered nurses navigate the transition.

## Data Availability

The datasets used and/or analyzed during the current study are available from the corresponding author upon reasonable request.
